# Moral Deference and the War in Gaza

**DOI:** 10.1007/s11673-025-10514-8

**Published:** 2026-01-29

**Authors:** Zohar Lederman

**Affiliations:** 1 Department of Emergency Medicine, LKS School of Medicine, University of Hong Kong, Hong Kong, China; 2 Centre for Medical Ethics and Law, University of Hong Kong, Hong Kong, China

**Keywords:** Gaza, Moral deference, Moral expertise, Ethicists

## Abstract

This paper discusses the idea of moral deference in the context of the ongoing war in Gaza. This idea is particularly pertinent where many ethicists seem to share the sense that the Israeli onslaught in Gaza is immoral, and yet only few articulate it in writing. Moral deference, I suggest, can be a temporary measure against such paralysis, by referring to international humanitarian law.

## Introduction

Ethicists are arguably experts in the analysis of moral problems and thus in moral judgment (Singer 1972; Gordon 2014; Niv and Sulitzeanu-Kenan 2022). In our role as individuals who care about morality, professional ethicists, and privileged academics for the most part, ethicists arguably have an obligation to address concrete and urgent injustices and moral dilemmas (Chomsky 1967). Bioethicists arguably have a parallel obligation to address concrete and urgent injustices and moral problems with any bearing on health and healthcare. Several recent publications urge (bio)ethicists to pay more attention to, and become experts in, armed conflicts for example even if they occur in distant geographical contexts (Degrazia 2025; Jecker, Atuire et al. 2025; Lederman 2025a, b; Lederman and Lederman 2025).

Yet, after fourteen (when this manuscript was first submitted) months of intense fighting in Gaza and obvious, severe violations of international humanitarian law and human rights, the silence of ethicists (Shekhani and Jafarey 2024) and—perhaps worse—their inaccurate portrayal of the relevant history and present affairs (Singer 2023; Wynia 2023, 2024; Porat-Wojakowski, Elbo Arama et al. 2025; Steinberg 2025)—are striking. The psychological reasons for this silent and unbefitting scholarship (I emphasize “unbefitting” as I am otherwise a fan of Singer and Wynia) are intriguing but will only be touched upon lightly. Here, however, I wish to focus on one argument that I have been hearing from colleagues in response to the question: “why aren’t you writing about the war in Gaza?”.

This argument, essentially, is that they do not know enough about it and that despite their wish to lament the severe violation of basic human rights, they simply do not know what to say. I shall call this argument “the argument from lack of expertise.” In what follows, I will present and defend one way ethicists can and indeed should overcome this hurdle thus engaging in more activist academic writing, both specifically regarding the current war and generally. Espousing the idea of moral deference, I will argue that international humanitarian law (IHL) may be used as moral guidance, while acknowledging that it is an imperfect guide that should continuously be evaluated in specific contexts (Lederman 2025a, b).

### Gaza and the Silence of Ethicists

Most colleagues who agree that the Israeli onslaught in Gaza is unjustified and even appalling have not written about it (obviously, writing and publishing are not the only kind of academic engagement but, as far as I am aware, what is said here also applies for these other possible engagements such as conferences. Some bioethicists have done podcasts, and the argument here probably does not apply to them). Why? Three kinds of explanatory reasons are usually provided by them. First, the war machine is running too fast, too many atrocities are occurring, and they simply cannot keep up—by the time they would write about X, Y would happen that is actually worse. Second, that they are academic bioethicists and not human rights advocates; as moral experts (Niv and Sulitzeanu-Kenan 2022), they write about ethical dilemmas arising in biomedical contexts, and there is really no ethical dilemma here. Third is the argument from lack of expertise. While a lot can be said about the first two arguments, here I focus on the latter.

The argument from lack of expertise can of course be a prudential argument in the sense that it merely expresses self-interest and is not meant to be generalizable. For the sake of discussion and for future generations of ethicists, however, here I will formalize it as a normative analytic argument.

The argument from lack of expertise, then, may roughly be formalized thus:

Premise I (implicit): Ethicists should only write about issues they are knowledgeable about.

Premise II: Some ethicists are not knowledgeable about the history of the Israeli occupation or the details of the war.

Conclusion: These ethicists who are not knowledgeable about the Israeli occupation should not write about the ongoing war.

This argument is valid. Question is—is it sound? My answer is mostly “no,” because the first premise is mostly false or at least misleading. Part of the reason premise I is false or misleading has been discussed elsewhere—we write what we know, but we should also know what we write (Lederman and Lederman 2025). We usually (and hopefully) write about issues that we are passionate about. This passion drives us to spend long hours in front of the computer or with a book. In fact, having the privilege to work on issues that we are passionate about is one of the great advantages of academia. Often this passion exists before we start writing, but sometimes it comes as we write or read or listen to a lecture about a topic. The point here is that if we force ourselves to read about a topic that we are initially not passionate about, the passion may ensue in due time. And whether we force ourselves to read about a topic depends, among other factors, on whether we decide we should care about it. Whether we care enough about a topic to read about it and hence potentially become passionate about it depends, in part, on our own conviction that we should care about it. We write about what we care about, and caring about something may sometimes mean convincing ourselves that we should care about it.

Premise I is false, or at least partially false, for another reason. There seems to be a mismatch between current topics and issues to write about and the capacity of ethicists. Novel technologies in healthcare, including AI, armed conflicts and other major human rights violations, deprivation of women’s reproductive rights, patients’ autonomy, emerging infectious diseases etc. all merit our immediate attention. At the same time, personal limitations, administrative work, non-research academic commitments, and the mere efforts needed to analyse an issue or dilemma, as well as publish mean that there is only so much we can do. Ethicists thus need to prioritize. Since becoming knowledgeable about, or an expert on any topic requires time (Singer 1972), the natural inclination is to write about issues that one is already an expert on. This, of course, means that one does not write about other issues, morally serious and urgent as they might be. After all—how can one possibly make moral judgments about X without being an expert on X? One answer is moral deference.

### Moral Deference

David Enoch has previously argued for deference to experts in forming ethical judgments in order to minimize the risk of forming misguided judgments and thus acting wrongly (Enoch 2014). He specifically referred to war:

Suppose that the war is indeed indefensible, I—erroneously thinking that it is justifiable—vote for funding it, innocents die, and a family member criticizes me for my vote. If I did all that I could (within certain constraints) to find out whether the war was morally defensible, and failed, at least I can explain that I was not certain about the moral status of the war and I did what I could to find out … Now suppose that Alon [who is a moral expert] told me before the vote that the war was indefensible. If so, it no longer seems that I can answer in good faith that I did what I could to find out about the moral status of the war. (Enoch 2014, 15)

Enoch also evaluated the claim that it would have been more praiseworthy if he had better informed himself and achieved autonomy and moral understanding of the issue in question rather than defer to the judgment of others. His reply is that it would have indeed been more praiseworthy in principle, but since moral understanding takes time, and more harm may be caused during this time, expediency permits and even encourages deference. Because of this and other potential reasons, it may generally be preferable to defer to moral experts rather than trying to gain a full moral understanding and autonomy.

At the same time, Enoch claimed that when the risks to the parties of making a wrong judgment are not equal, it may be advisable to err on the side with less significant risks and suspend judgment, so as to minimize the harm caused by wrongdoing.

In mid-November 2023, in response to some ethicists’ call for a ceasefire, Enoch argued that the war in Gaza is morally complex and so is the ethics of commenting on it. Nevertheless, he acknowledged two undeniable truths: that what Hamas did was horrendous and that many Gazans are innocent. He also claimed that Israel has a right to defend itself and a duty to protect its citizens. In light of these right and duty, and consistent with his claim above, Enoch was careful not to call for a ceasefire—

And if you think that a call for a ceasefire is justified, given factual uncertainties, as a way of erring on the side of safety, you should remember that when it comes to war, often there is no side of safety. Any mistakes—using too much force, not using enough—will be paid for in the only relevant currency, the blood of innocents. (Enoch 2023)

Lastly, Enoch himself admits that the Israeli government has long lost the trust of many, including himself, but still warns public intellectuals about encouraging wrongful policies: “When public intellectuals make confident but factually unfounded proclamations on such matters, they degrade their respective fields, and to the extent that they have an effect in the real world they risk taking part in bringing about disastrous policies” (Enoch 2023). These remarks were heavily criticized^1^ as supportive of genocide—a clearly unfair critique.

In mid-March, 2024, Enoch claimed that he no longer supports the war in Gaza. While initially justifiable, *inter alia* to reinstate the power of deterrence against Hamas and other hostile forces in the region (which, according to him, saves lives on both sides) the war has now achieved the maximum deterrent effect, and the Israeli government now seems to be indifferent to the lives of innocent Gazan civilians and even Israeli hostages and soldiers. Under these circumstances, the international community should forcefully strive to stop the fighting (Enoch 2024).

Similarly, I argue that it may at times be preferable to defer to international humanitarian law (IHL) and human rights law (hereon this will be subsumed under IHL for convenience) as ethical guidelines with an ethical authority, until one is able to form a more considered moral judgment. In other words, deference not to the experts themselves but to guidelines written by (legal and) moral experts may be the right thing to do in real life situations when one is expected to form a decisive, educated opinion about an ongoing event. Philosophers would of course be aghast about the potential logical fallacy of appeal to authority (Matheson, McElreath et al. 2018) and even worst, an is-ought gap whereby we rely on law to make ethical judgments. To them I say, following Enoch, that the price of not forming and voicing an opinion—even without full moral understanding—may have worse consequences. Furthermore, there are other reasons to justify the reliance on IHL.

### IHL and its Moral Foundations

At least two reasons may plausibly justify the moral authority of IHL. First, as normative *legal* guidelines, they are likely to be largely correct. Contemporary IHL has a rich philosophical, legal, and historical tradition, both in the United States and Europe. In the American context, it traces back to the “Instructions for the Government of Armies of the United States in the Field” prepared by Francis Lieber, a jurist and political philosopher, in 1863. The Lieber Code (Lieber 1863) was commissioned by the U.S. army to govern the conduct of the Union Army during the American Civil War and approved by the American President. Compared to Henry Dunant’s work—its European counterpart—the Lieber Code prioritized the interests of one’s own country to a much larger extent, even allowing for the starvation of enemy combatants:

Article 17: “War is not carried on by arms alone. It is lawful to starve the hostile belligerent, armed or unarmed, so that it leads to the speedier subjection of the enemy.”

But current IHL has moved away from such emphasis on one’s country to the detriment of the enemy party. Particularly in the form of the four Geneva Conventions and the two Additional Protocols which were written across five decades, as well as the extensive Commentary associated with them, the current IHL was carefully drafted. Its authors were capable jurists who devoted significant time and effort in drawing from historical documents and experiences, distilling and articulating the principles of humanity that ought to guide and curb offensive and distrustful human behaviour. And they had to do it under immense political pressures, often pulling in opposing directions (Lederman, Irfan et al. 2025).

Yet principles of humanity they indeed are. The second reason that may plausibly support the moral authority of IHL is its content. While allowing for the intentional starvation of enemy combatants, even the Lieber Code captures the inviolable moral value of all humans, even those bearing arms on the enemy side: “Men who take up arms against one another in public war do not cease on this account to be moral beings, responsible to one another, and to God” (Lieber 1863, 8). IHL takes the right to life for granted, “for without it there would be no reason for the other rights mentioned” (Pictet, Uhler et al. 1958, 201). In a much more elaborate fashion compared to the Lieber Code, IHL is explicitly grounded in provisions that ethicists would immediately recognize as maxims in their own work.

One such provision is Respect for the Person, namely to physical, moral, and intellectual integrity:

The right of respect for the person must be understood in its widest sense : it covers all the rights of the individual, that is, the rights and qualities which are inseparable from the human being by the very fact of his existence and his mental and physical powers; it includes, in particular, the right to physical, moral and intellectual integrity-an essential attribute of the human person. (Pictet, Uhler et al. 1958, 201)

Another absolute provision is Respect for Honour, which bans degrading treatment of civilians including insults:

Honour is a moral and social quality. The right to respect for his honour is a right invested in man because he is endowed with a reason and a conscience. The fact that a protected person is an enemy cannot limit his right to consideration and to protection against slander, calumny, insults or any other action impugning his honour or affecting his reputation; that means that civilians may not be subjected to humiliating punishments or work. (Pictet, Uhler et al. 1958, 202)

These and other provisions in the Geneva Conventions and their Commentaries, such as the emphasis on human dignity, would surely find wide agreement among ethicists who themselves tend to perceive these as maxims (while perhaps admitting they are vague and in need of specification). This convergence affirms that these moral commitments are highly intuitive and suggests that their implications as expressed in the Articles of the Geneva Conventions are at least prima facie authoritative.

### IHL in Gaza

There seems to be a wide agreement in international law and among international law experts (Gardam 2004; Steinhoff 2007; Erakat 2019) that *jus ad bellum* depends on *jus in bello.* In other words, even if the purpose of a war is just and engaging in war is necessary and the risks are proportional to the potential benefits, thus making it a just war, the way in which a war is fought is also a necessary condition to whether a war is just. If the conduct of war is unjust, the war becomes unjust, even if the decision to wage it adhered to the principles of necessity and proportionality.^2^

Now, to be fair (and after discussing this with Enoch), all wars are expected to involve acts that are considered to be unjust. This means that if we were to simplistically abide by the principle that every unjust act during war means that the war itself becomes unjust we would, for all purposes, become pacifists. Instead, there must be a threshold of unjust acts beyond which a war itself becomes unjust, and this could be evaluated based both on the quality and quantity of the acts committed (Gross 2006). One could argue that some acts may be so egregiously wrongful that even a single occurrence would entail that the war as a whole is unjust—the bombardment of hospitals while no enemy combatant is actively shooting at you (meaning, while not in immediate self-defence) comes to mind (Lederman 2025a, b). This position, however, is unlikely to sit well with most just war theorists. Conversely, some acts may not merit a wholesale denunciation of the war. What we need are criteria to evaluate when unjust acts amount to such injustice that crosses the threshold.

While I do not pretend to provide such criteria here, it might be useful to consider the use of White Phosphorus (WP) as an example. It is used first to inform the reader who may not be aware of the relevant history and second to evaluate our intuitions regarding the point in which unjust acts such as use of WP make a just war unjust.

Already in October 2023 evidence was amounting that the Israeli military was again using WP airbursting in Gaza (and Southern Lebanon) (Christou, Horton et al. 2023). White Phosphorus is an incendiary substance that can cause severe burns, respiratory distress, and death. Victims of WP and healthcare professionals caring for these patients report severe pain and prolonged suffering and death without immediate specialist treatment. Dressed wounds may re-ignite upon exposure to atmospheric oxygen (Human Rights Watch 2023). Even if they survive, patients typically require intensive long-term care including plastic surgery and extensive debridement, infection control and complex fluid management. The effects of WP on children are even more severe than effects on adults, as they impair normal physiological growth. In addition to causing severe pain and long term disability, the use of WP is indiscriminate, as its damage radius is impossible to control (Human Rights Watch 2009, 2020). Regardless of whether WP is intended for use for illumination, its use in a densely populated area such as the Gaza Strip is illegal under international law (Human Rights Watch 2009, 2023).

This was not the first time Israel has used WP, and a short historical digression may be instructive.

### The Russell Tribunals

Bertrand Russell was particularly concerned about the use of excessive fire power by the U.S. military in Vietnam, including their use of chemical and incendiary weapons. He followed the history of similar tribunals in the past and established the International War Crimes Tribunal in Stockholm. The tribunal was intentionally fashioned after the Nuremberg trials. In a decision that likely galvanized the anti-war movement in the United States, the United States was found guilty of war crimes, and in his concluding remarks Russell claimed that one hundred thousand tons of napalm were never comparable to a peasant holding a gun (Klinghoffer and Klinghoffer 2002; Mehta 2012). The Russell tribunal cleared the path for future tribunals in the form of Permanent People’s Tribunals (PPT).

The Arab League requested a PPT in 1967 but was rejected after Jean-Paul Sartre and Simone de Beauvoir, who were members, opposed the request (Klinghoffer and Klinghoffer 2002, note 50, 237). This particular PPT, otherwise known as the Russell Tribunal on Palestine (RToP), was finally established in 2009, in response to Operation Cast Lead. The Tribunal found that the fifty tonne munition used by Israel in Gaza was equal in seismic power to a small earthquake, and that WP was used by Israel (Russell Tribunal on Palestine 2014). Human Rights Watch (HRW) in turn

… found no evidence that Israeli forces fired ground-burst white phosphorous at hardened military targets, such as Palestinian fighters in bunkers, but it may have used air-burst white phosphorous for its incendiary effect. Fired from artillery and air-burst to maximize the area of impact, white phosphorous munitions will not have the same lethal effect as high-explosive shells, but will be just as indiscriminate … (2009, 1–2)

Israel at first denied this latter allegation but soon thereafter admitted it. After the policy permitting use of WP by the Israeli military was challenged at the Israeli High Court, the Israeli military declared that it would no longer use the chemical except for two extraordinary scenarios, which were privately disclosed to the court. These exceptions still remain unknown to the public.

An emergency session of the RToP took place 2014 in response to operation Protective Edge. This Tribunal found Israel guilty of war crimes and crimes against humanity (2014). Having dropped four hundred tons of bombs, the Israeli military was found guilty of “use of weapons, projectiles, and material and methods of warfare which are of a nature to cause superfluous injury or unnecessary suffering or which are inherently indiscriminate …” (2014, 6). The Tribunal found that WP had been used but in a “limited capacity” (The Tribunal specifically criticized the Dahiya Doctrine adopted by the Israeli military, which effectively permits the disproportionate killing of Palestinian citizens, supposedly as a deterrent to further liberatory action).

Then, same as now, questions of *Jus ad bellum*—the justification for entering into war—have been scrutinized. But in the context of war, the conditions of *jus in bello*—the laws governing the conduct of war—must be respected. Both Amnesty International and HRW have claimed that the use of WP by Israel is disproportionate, indiscriminate, and illegal. Citizens and combatants alike should never be subjected to WP. Israel has already been condemned for using WP in Palestine and should never have been afforded the impunity to use it again. Its use in congested residential areas clearly constitutes a breach of the 1980 Convention on Certain Conventional Weapons (International Committee of the Red Cross 2003). Even in times of war, basic moral principles and rules apply, i.e. respect for body integrity and honour, and the derivative obligation to prevent inhumane and unnecessary harm to others.

But again, taking all of this under consideration, and from a just war theory perspective—does the use of WP by Israel automatically make the war in Gaza unjust *ad bellum*?

This is an intriguing philosophical question that should be explored further, abstractly. In the context of the ongoing violence—as admitted by Enoch—any claim to engaging in just war in Gaza, or any attempt to blame Hamas for the murder and starvation of unarmed civilians, is nothing but a farce. Regardless of whether the use of WP crosses the threshold to make an originally just war unjust, the use of WP is not the only way in which Israel has egregiously violated IHL during the ongoing violence (United Nations 2024). And this was true one month into the war, let alone after fourteen months. In fact, Israel has been breaking IHL much before October 7th (ICJ 2004). Even if bioethicists know nothing about the history of the specific conflict, they can rely on IHL, reflected for instance in decisions by International Courts (ICJ 2004, 2024, ICJ 2024), to publicly criticize and condemn the Israeli government’s conduct and demand justice for Palestinians.

## Concluding Remarks

What does all of the above mean for ethicists? Deference to moral expertise may indeed be justified at times, and such moral expertise may also be found in international legal instruments. These legal instruments also negate a claim made by Enoch—that Israel has a right of self-defence. Israel is an occupying power, that even after 2005 maintained effective control over Gaza, be it in land, air, or sea. As such, it actually does not have a right of self-defence according to international law (Erakat 2019) and the International Court of Justice (ICJ 2004). Quite to the contrary, Israel has an obligation to sustain the population under its control, which it has repeatedly failed to do (Erakat 2019; Lederman, Lederman et al. 2022).

In summary, deference to moral expertise is permissible, but it is also fishy (Enoch 2014). For ethicists, there is something particularly fishy about relying on legal instruments to make moral judgments. So by all means, if ethicists have time, they should try to achieve moral understanding and autonomy before making a specific moral judgment. Alternatively, they may continue to deliberate and evaluate their moral judgment after it was made and implemented (Matheson, McElreath et al. 2018). But when so many ethicists seem to be (understandably) paralyzed when it comes to denouncing the onslaught in Gaza, the minimum they could do is to cite international law and empirically and analytically demonstrate the ways in which Israel repeatedly violates it. Alternatively, they can rely on meticulous analyses conducted by legal experts (2024) and unoriginally augment the voices of the un-paralyzed.

## Data Availability

Not applicable

## Footnotes

1 Abdulsattar X. 2024. An disingenuous attempt at justifying Palestinian genocide: A response to professor David Enoch. *Medium*, Jan 20. https://medium.com/@abdulsattarx/an-disingenuous-attempt-at-justifying-palestinian-genocide-a-response-to-professor-david-enoch-d77d1a438524. Accessed April, 2024.

2 A reviewer disagrees with his claim, arguing instead that *jus ad bellum* continues to apply throughout the course of the war.
